# Intermittent administration of peracetic acid is a mild environmental stressor that elicits mucosal and systemic adaptive responses from Atlantic salmon post-smolts

**DOI:** 10.1186/s40850-021-00100-x

**Published:** 2022-01-04

**Authors:** João Osório, Kevin T. Stiller, Britt-Kristin Reiten, Jelena Kolarevic, Lill-Heidi Johansen, Fernando Afonso, Carlo C. Lazado

**Affiliations:** 1 CIISA, Faculty of Veterinary Medicine, University of Lisbon, 1300-477 Lisbon, Portugal; 2 Nofima, The Norwegian Institute of Food, Fisheries and Aquaculture Research, 1433 Ås, Norway; 3 Nofima, The Norwegian Institute of Food, Fisheries and Aquaculture Research, 6600 Sunndalsøra, Norway; 4 Nofima, The Norwegian Institute of Food, Fisheries and Aquaculture Research, 9019 Tromsø, Norway

**Keywords:** Disinfection, Nasal immunity, Mucosal immunity, Oxidative stress, RAS

## Abstract

**Background:**

Fish encounter oxidative stress several times during their lifetime, and it has a pervasive influence on their health and welfare. One of the triggers of oxidative stress in fish farming is the use of oxidative disinfectants to improve rearing conditions, especially in production systems employing recirculation technology. Here we report the physiological and morphological adaptive responses of Atlantic salmon (*Salmo salar* L.) post-smolts to intermittent exposure to a potent oxidative agent peracetic acid (PAA). Fish reared in semi-commercial scale brackish water recirculating aquaculture system (RAS) were exposed to 1 ppm PAA every 3 days over 6 weeks. Mucosal and systemic responses were profiled before exposure, 22 and 45 days during the intermittent PAA administration.

**Results:**

Oxidative stress was likely triggered as plasma antioxidant capacity increased significantly during the exposure period. Adaptive stress response to the periodic oxidant challenge was likewise demonstrated in the changes in plasma glucose and lactate levels. PAA-induced alterations in the transcription of antioxidants, cytokines, heat shock proteins and mucin genes showed a tissue-specific pattern: downregulation was observed in the gills and olfactory rosette, upregulation occurred in the skin, and no substantial changes in the liver. Further, PAA exposure resulted in histological changes in key mucosal organs (i.e. olfactory rosette, skin and gills); pathological alterations were predominant in the gills where cases of epithelial lifting, hypertrophy and clubbing were prevalent. In addition, intermittent PAA administration resulted in an apparent overproduction of mucus in the nasal mucosa. Lastly, PAA did not dramatically alter the ability of salmon to mount a physiological stress response in the presence of a secondary stressor, though some subtle interference was documented in the kinetics and magnitude of plasma cortisol and glucose response post-stress.

**Conclusions:**

The present study collectively demonstrated that intermittent oxidant exposure was a mild environmental stressor that salmon could mount strong adaptive responses at systemic and mucosal levels. The results will be valuable in optimising the rearing conditions of post-smolts in RAS, especially in adopting water treatment strategies that do not considerably interfere with fish health and welfare.

**Supplementary Information:**

The online version contains supplementary material available at 10.1186/s40850-021-00100-x.

## Background

The Atlantic salmon (*Salmo salar* L.) is one of the world’s major farmed fish species. Norway supplies almost 50% of the global salmon production, thus playing a vital role in the economy through value creation, employment and tax revenues [1]. In 2018, Norway’s total aquaculture production reached 1,354,941 t [2]. However, the long-term growth of the industry is threatened by multiple challenges such as prolonged low-temperature periods making year-round intensive aquaculture production challenging, high prevalence of sea lice (*Lepeophtheirus salmonis*) infestation, escapees, increasing concerns regarding wastewater management and environmental footprints, and animal welfare [3].

In recent years, a significant effort has been dedicated to addressing these challenges by developing solutions that will enable better control of the production environment. Recirculation aquaculture systems (RAS), comprised of multiple units, including culture tanks, mechanical and biological filtration, oxygenation and degassing, have been identified as a potential solution to tackle these challenges [4, 5]. Adoption of RAS offers multiple advantages compared with traditional smolt production in flow-through systems [6], as it allows a more flexible location of the production sites, water conservation, more efficient waste management and nutrient recycling, enhanced biosecurity and disease control, prevention of escapees, and reduced susceptibility to challenging and erratic environmental conditions [4, 7]. Active efforts are currently being undertaken in producing post-smolts in RAS, though a number of biological issues need to be addressed [4, 8]. Since fish production in RAS is generally conducted in high densities, with long water retention times and high feeding rates that promote high organic loads and micro-particle accumulation, favourable conditions for opportunistic bacterial growth may arise. The risk for pathogenic bacterial accumulation in the system is considerable [9, 10]; hence, the system must secure effective biosecurity measures. Thus, routine disinfection is a crucial component of the system.

Peracetic acid (PAA) is a strong oxidative disinfectant and commercially available as a quaternary equilibrium mixture of PAA, acetic acid, hydrogen peroxide (H_2_O_2_), and water. PAA is one of the disinfectants approved for aquaculture use in Norway, though the application is limited as a surface disinfectant (www.mattilsynnet.no). Disinfection is mainly achieved by releasing oxygen radicals, causing oxidative disruption of cell membranes [11–14]. PAA is regarded as a promising disinfectant for improving biosecurity in aquaculture due to its broad spectrum of activity against several microorganisms, short contact time, low dependency on pH, and rapid degradation into harmless residues [15–18]. It is also identified as an alternative to H_2_O_2_ since it degrades faster and presents a lower effective dose against many pathogens (1-2 mg L^− 1^) than H_2_O_2_, making it safer for the biofilter and therefore more suitable for application in RAS [19–21].

Even though the toxicity of PAA towards various fish species has been documented [22, 23], the current knowledge about the physiological impacts of PAA-based disinfection remains fragmentary. Most studies focused on the physiological effects of PAA routine disinfection are in rainbow trout (*Oncorhynchus mykiss*). It was demonstrated that therapeutic doses of PAA (0.2 – 1.4 mg L^− 1^) could trigger immunological and stress responses in trout raised in RAS [24–26]. Intermittent application of PAA triggered oxidative stress in trout, as indicated by an increase in circulating free radicals for which the fish counteracted by mobilising essential antioxidants [26]. Moreover, it was demonstrated that PAA-exposed trout could mount a normal physiological stress response to a secondary stressor supporting it as a welfare-friendly antimicrobial agent [25]. PAA has recently been evaluated in salmon smolts as a bath chemotherapeutant, and the results revealed that the fish were able to mount mucosal and systemic responses to PAA exposure at different therapeutic doses (0.6 to 4.8 ppm) [27]. It is yet to be shown how salmon would respond when exposed to the oxidant intermittently over a prolonged period. Intermittent exposure is a relevant and practical PAA application protocol for salmon in RAS, as efficient disinfection is achieved with limited logistical input.

Here we present the physiological and morphological impacts of intermittent PAA oxidant exposure in Atlantic salmon post-smolts reared in a semi-commercial scale brackish water RAS. We employed gene expression, biochemical assays and quantitative histology to assess how the health and welfare of salmon were influenced and shaped by PAA exposure. To test the hypothesis that intermittent exposure does not impair salmon responses to a secondary stressor, we performed a handling-confinement stress test before and after intermittent PAA administration.

## Results

### Production performance and external welfare indicators

There was no recorded treatment-related mortality during the PAA administration period. Moreover, daily visual inspection revealed no considerable changes in feeding behaviour. The average weight at termination was 280 ± 8 g (mean ± SD), accounting for a mean weight gain of 166 ± 9.4 g and a specific growth rate of 2.0 ± 0.2%/day.

External welfare scoring focused on four key external indicators – skin and fin damages (including dorsal, caudal, pectoral and pelvic fins) (Supplementary file 1). There was a significant difference in the prevalence of skin damages on the left side of the fish between before exposure and day 45, where cases increased significantly following intermittent exposure. Such a significant change on the skin was not identified on the right side of the fish. On day 45, 90% of the skin damages at both sides were scale loss, while the remaining 10% accounted for minor haemorrhaging cases. Dorsal, caudal, pectoral and pelvic fin damages revealed no significant alterations before and after the PAA administration, and around 80% of the recorded cases were active damages (i.e. splitting).

### Plasma stress parameters of oxidant-exposed salmon post-smolts

**Fig. 1:**
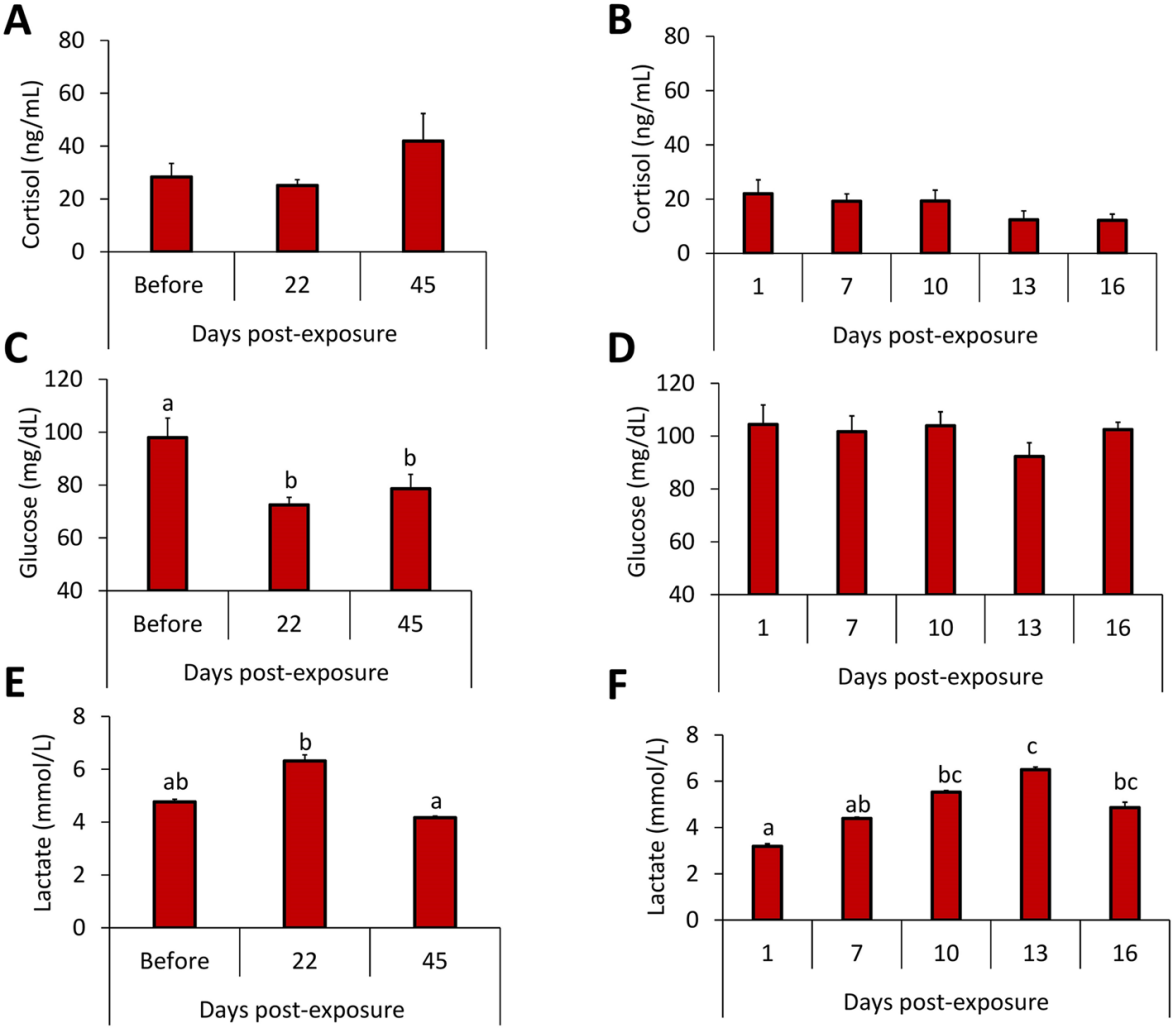
Plasma levels of key stress response indicators (i.e. cortisol, glucose and lactate) in Atlantic salmon intermittently exposed to PAA. The right panel (**A**, **C**, **E**) displayed the levels in the samples collected during the 3 major sampling points (before, and at days 22 and 45 of intermittent exposure), whereas the left panel (**B**, **D**, **F**) displayed the measured levels in the samples collected during the first 2 weeks after the start of the intermittent application. Values presented are mean ± SE of 8 individual fish per sampling point (2 fish per replicate tank). Different letters denote significant difference at *P* < 0.05

There were no significant temporal changes in the plasma cortisol levels during the 3 major sampling points of the exposure trial (Fig. 1A). Similarly, no significant differences were found in the plasma cortisol levels during the first 2 weeks of intermittent exposure (Fig. 1B). Plasma glucose levels showed a significant 26 and 20% decrease at days 22 and 45 during the intermittent exposure, respectively, relative to the level before oxidant administration (Fig. 1C). During the first 2 weeks of intermittent exposure, plasma glucose levels were stable (Fig. 1D). Plasma lactate levels at days 22 and 45 displayed no significant differences with the pre-exposure level (Fig. 1E). However, there was a significant difference in the plasma lactate level between days 22 and 45 post-exposure, where a decrease of about 34% was identified. An increasing tendency was observed in the plasma lactate levels during the first 2 weeks of exposure (Fig. 1F). The levels from day 10 onwards were significantly different from the level observed 1 day after the start of the intermittent application, where a fold increase was identified.

**Fig. 2:**
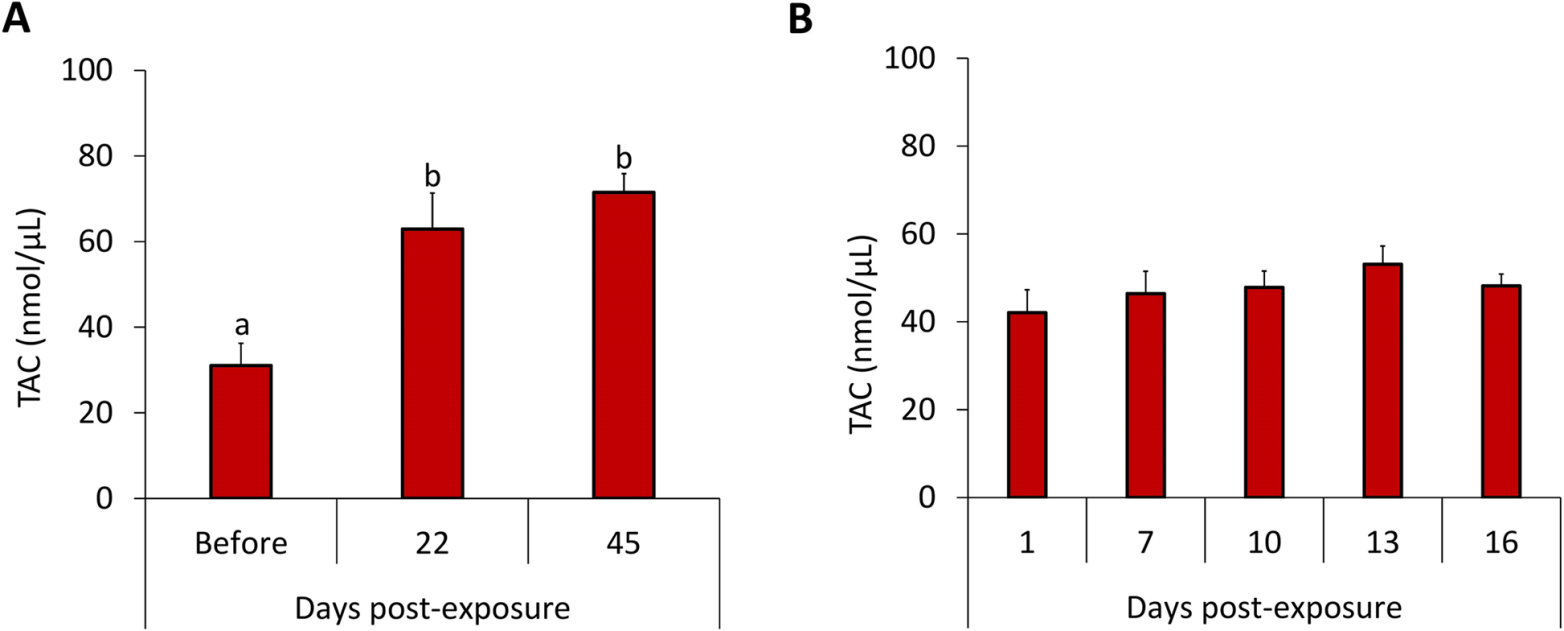
Total antioxidant capacity (TAC) in the plasma Atlantic salmon intermittently exposed to PAA. **A** TAC level in the samples collected during the 3 major sampling points (before, and 22 and 45 days after exposure), and **B** TAC level in the samples collected during the first 2 weeks after the start of the intermittent application. Values presented are mean ± SE of 8 individual fish per sampling point (2 fish per replicate tank). Different letters denote significant difference at P < 0.05

The total antioxidant capacity (TAC) in the plasma increased by at least one-fold at days 22 and 45 post-exposure, compared with the level before administration (Fig. 2A). No significant differences in the TAC levels were found during the first 2 weeks of exposure (Fig. 2B).

### Mucosal and hepatic expression of selected immune and stress-related genes

**Fig. 3:**
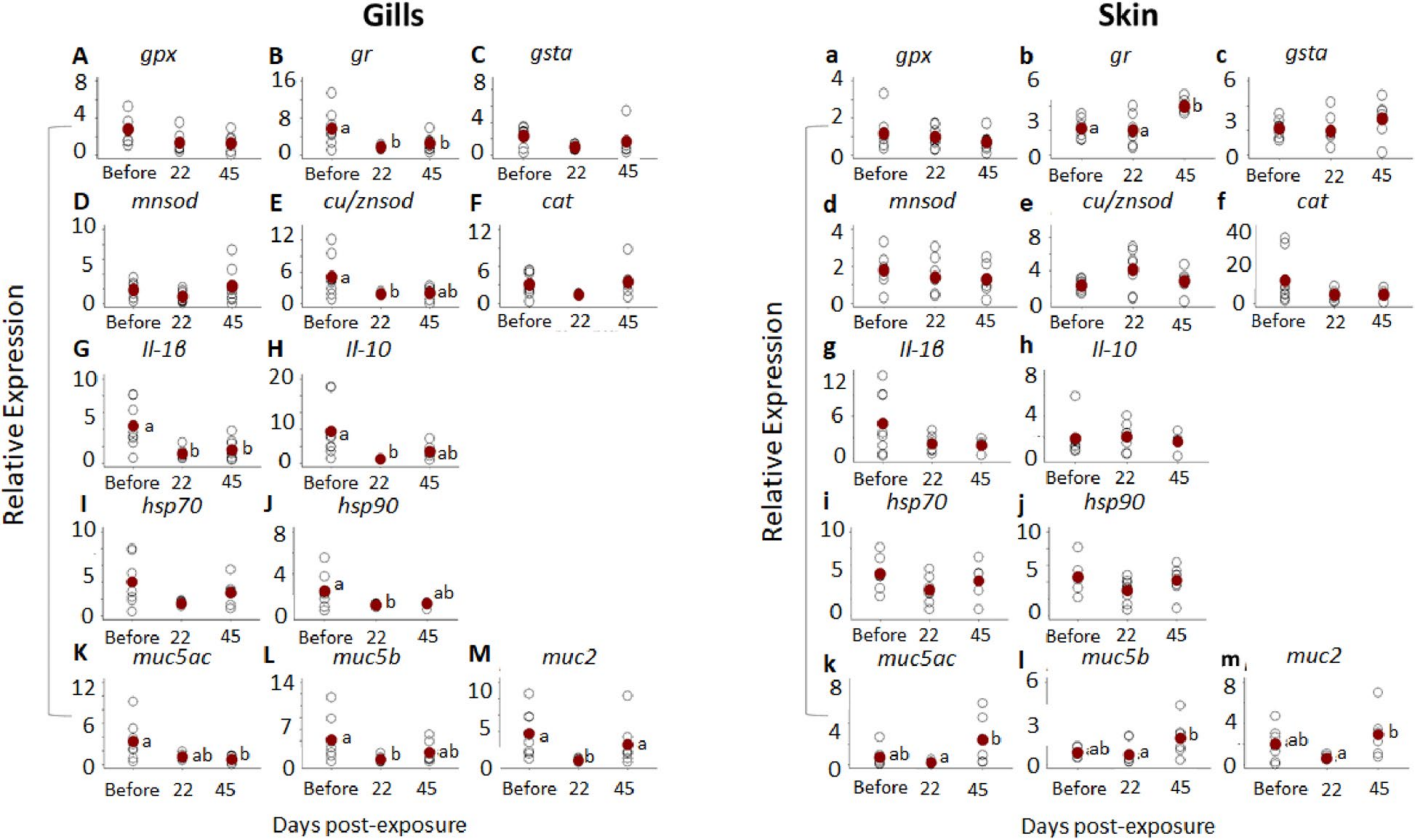
Expression of selected genes in the gills (A → M) and skin (a → m) of Atlantic salmon intermittently exposed to PAA. Each unshaded circle represents the expression of a particular gene in a single fish. The shaded circle denotes the mean expression value from 8 individual fish per sampling point. Samples were collected before and 22 and 45 days after intermittent exposure

The transcript levels of 13 selected immune and stress-related genes were quantified in the gills (Fig. 3A-M) and the skin (Fig. 3a-m) of salmon intermittently exposed to PAA. From the group of antioxidant defence genes, the expression of *glutathione reductase* (*gr*, Fig. 3B) and *copper/zinc superoxide dismutase* (*cu/znsod,* Fig. 3E) in the gills was significantly modulated by intermittent oxidant exposure - the expression of *gr* at both timepoints was significantly lower compared with the pre-exposure level. In comparison, the expression of *cu/znsod* was also significantly lower but only at day 22 post-exposure relative to pre-exposure. The expression profile of the same group of antioxidant genes in the skin revealed that only the expression of *gr* (Fig. 3b) was significantly affected by intermittent oxidant exposure. The transcript level at day 45 was significantly higher than the expression before and 22 days after intermittent administration.

The expression of 2 cytokines (i.e. *interleukin 1β, il1β* and *interleukin 10*, *il10*) was significantly affected by intermittent oxidant exposure in the gills (Fig. 3G, H) but not in the skin (Fig. 3g, h). The branchial transcription of both cytokines was significantly downregulated at day 22 relative to the pre-exposure level. Such significant downregulation was still persistent for *il1β* at day 45 of intermittent exposure (Fig. 3G).

The branchial *heat shock protein 90* (*hsp90*) expression was significantly affected by intermittent PAA exposure (Fig. 3J), where a downregulation was identified at day 22 compared with the pre-exposure level. The expression in the gills of the other heat shock protein gene, *heat shock protein 90* (*hsp70*), was not affected by intermittent oxidant exposure (Fig. 3I). Moreover, the expression of both *hsp*s was not significantly affected in the skin.

The transcription of all three mucin genes in both mucosal tissues was significantly affected by intermittent PAA exposure (Fig. 3K, L, M, k, l, m). The transcript level of *mucin 5 ac-like* (*muc5ac*) was significantly lower in the gills at day 45 than the level before the oxidant administration (Fig. 3K). The skin counterpart was identified to have a significantly higher expression at day 45 than at day 22 of intermittent administration, but not at the pre-exposure level (Fig. 3k). The *mucin 5b-like* (*muc5b*) expression in the gills at day 22 was significantly lower than the level before exposure (Fig. 3L), and a similar trend was likewise identified for *muc2* (Fig. 3M). There was a significant difference in the expression of *muc5b* in the skin between day 22 and 45; nonetheless, the levels were not significantly different from pre-exposure (Fig. 3l). The same expression pattern was observed for *muc2* (Fig. 3m).

**Fig. 4:**
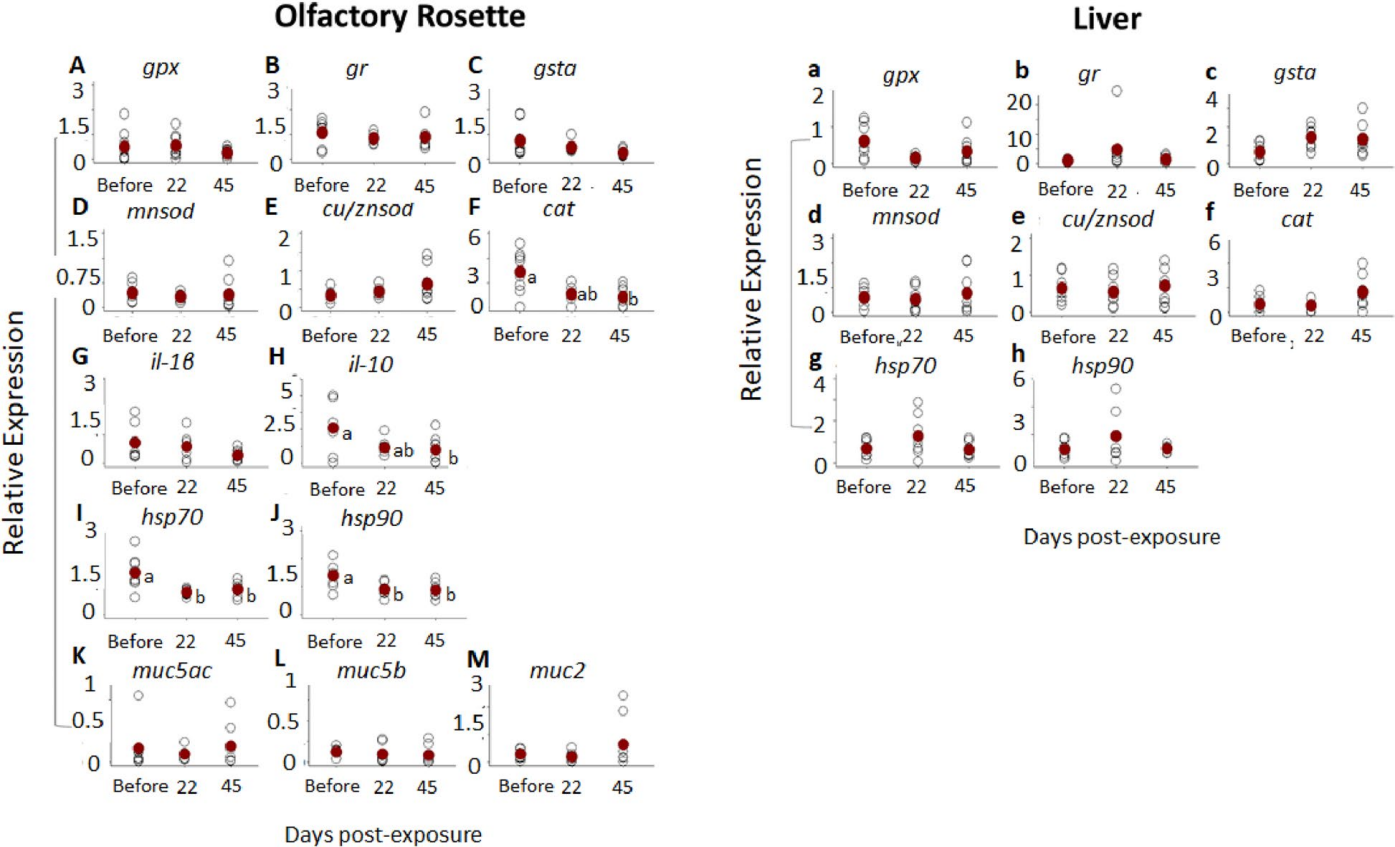
Expression of selected genes in the olfactory rosette (A → M) and liver (a → h) of Atlantic salmon intermittently exposed to PAA. Please refer to Fig. 3 for additional information

In the olfactory rosette, the expression of *catalase* (*cat*) was significantly lower at day 45 compared with the level before PAA administration (Fig. 4F), but no significant change was identified between mid- and termination samplings. The transcription of the rest of the antioxidant defence genes did not significantly vary during the exposure trial (Fig. 4A-E). There was a significant downregulation in the expression of *il10* at day 45 of intermittent exposure relative to the level before the oxidant application (Fig. 4H). On the other hand, the nasal expression of *il1β* was not significantly altered during the trial (Fig. 4G). Both genes coding for heat-shock proteins were affected considerably by intermittent oxidant exposure (Fig. 4I, J), where the expression of *hsp70* and *hsp90* was downregulated at days 22 and 45 of intermittent exposure relative to the before exposure level. None of the mucin genes was significantly affected in the olfactory rosette by intermittent oxidant exposure (Fig. 4K-M).

The expression of all investigated marker genes in the liver was not affected by intermittent oxidant exposure (Fig. 4a-h).

### Structural and morphometric changes in the mucosal organs following intermittent oxidant exposure

**Fig. 5:**
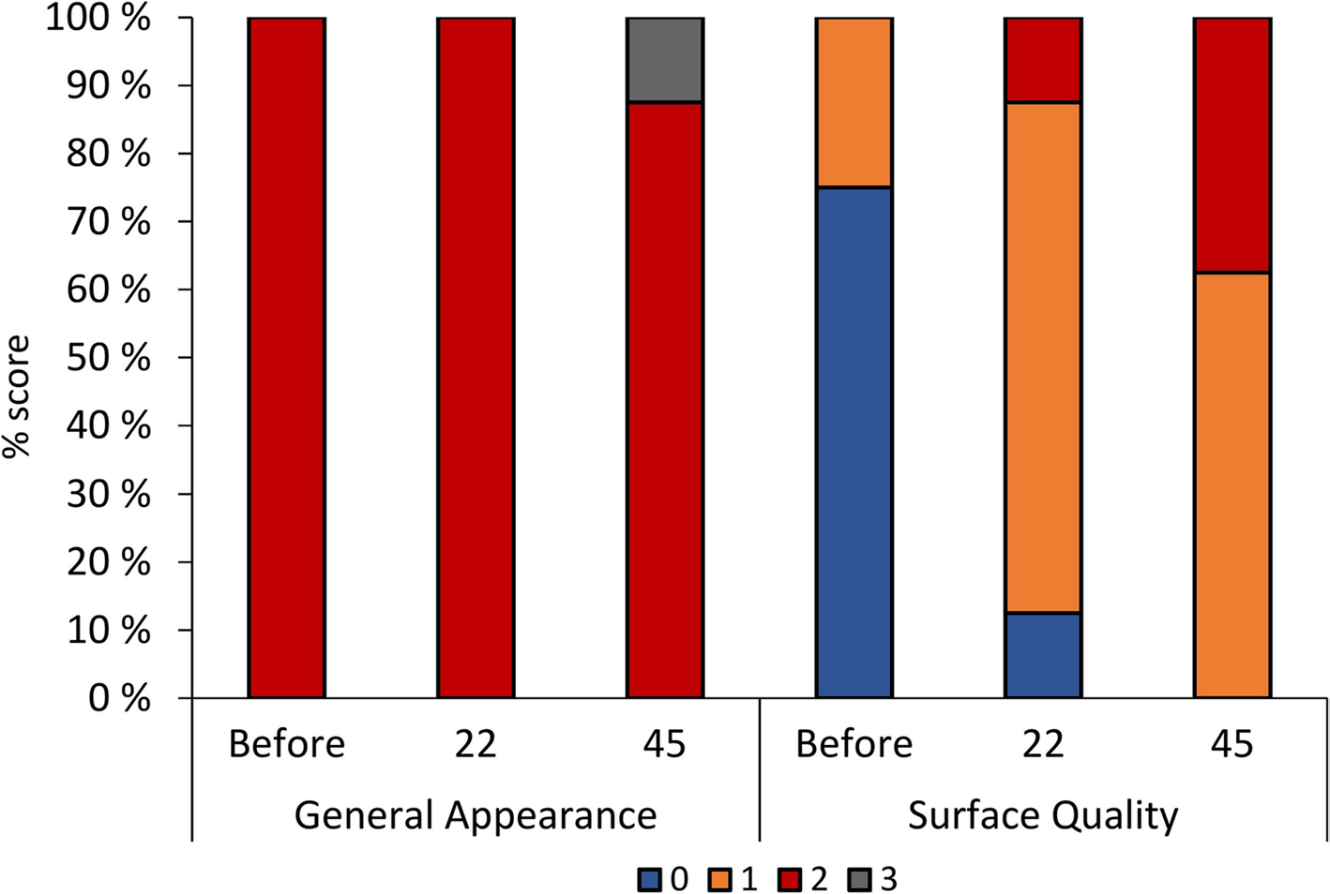
Distribution of skin health scores of Atlantic salmon intermittently exposed to PAA. Two key skin health parameters were evaluated: a) general appearance; b) epithelial surface quality. The 0-to-3 scoring was used, where 0 indicates good condition while 3 denotes severely compromised state. For detailed information on the scoring scheme, please refer to Stiller et al., 2020 [28] and Lazado et al., 2020a [29]. *N* = 8, number of fish analysed per timepoint

Key morphometries of the skin, including epidermal and dermal thickness, were differentially affected by intermittent oxidant exposure (Table 1). Although no significant differences were observed in the epidermal thickness, a marked dermal thickening was observed at days 45 and 22 of intermittent oxidant exposure. No significant differences were found in the total number of mucous cells and the distribution of acidic and neutral mucous cells. A semi-quantitative skin health scoring revealed no significant differences in the general appearance of the epidermis following intermittent oxidant exposure, though all recorded cases were 2 and above (Fig. 5). On the other hand, epidermal surface quality exhibited significant change through time, and cases with a score 2 were highest at day 45.

**Table 1:** Morphometric features of the skin and gills of Atlantic salmon intermittentally exposed to PAA

Tissue	Parameters	Days post-exposure		
		0	22	45
**Skin**	Epidermal thickness	38.5 ± 3.6 μm	39.7 ± 6.2 μm	50.6 ± 3.4 μm
	Dermal thickness	136.9 ± 5.6 μm^a^	127.9 ± 4.8 μm^a^	180.1 ± 7.3 μm^b^
	Number of mucous cells^a^	18.8 ± 1.8 (16.8/2)	21.6 ± 3.8 (19.6/2)	27.2 ± 2.4 (24.6/2.5)
**Gills**	Interlamellar space	31.6 ± 0.7 μm^a^	31.8 ± 0.8 μm^a^	26.6 ± 0.6 μm^b^
	Lamellar length	125.3 ± 3.8 μm^a^	140.5 ± 3 μm^b^	160.4 ± 2.4 μm^c^
	Number of mucous cells^a^	42.9 ± 4^a^ (37.4^a^/5.5)	83 ± 6.8^a^ (76.6^a^/6.3)	88.9 ± 10.1^b^ (84.3^b^/4.6)

^a^First number indicates the total number of mucous cells, while numbers inside the parentheses show the ratio of acidic (first) and neutral mucous cells (second). Different letters indicate significant difference. *N* = 8, number of fish analysed per timepoint

**Fig. 6:**
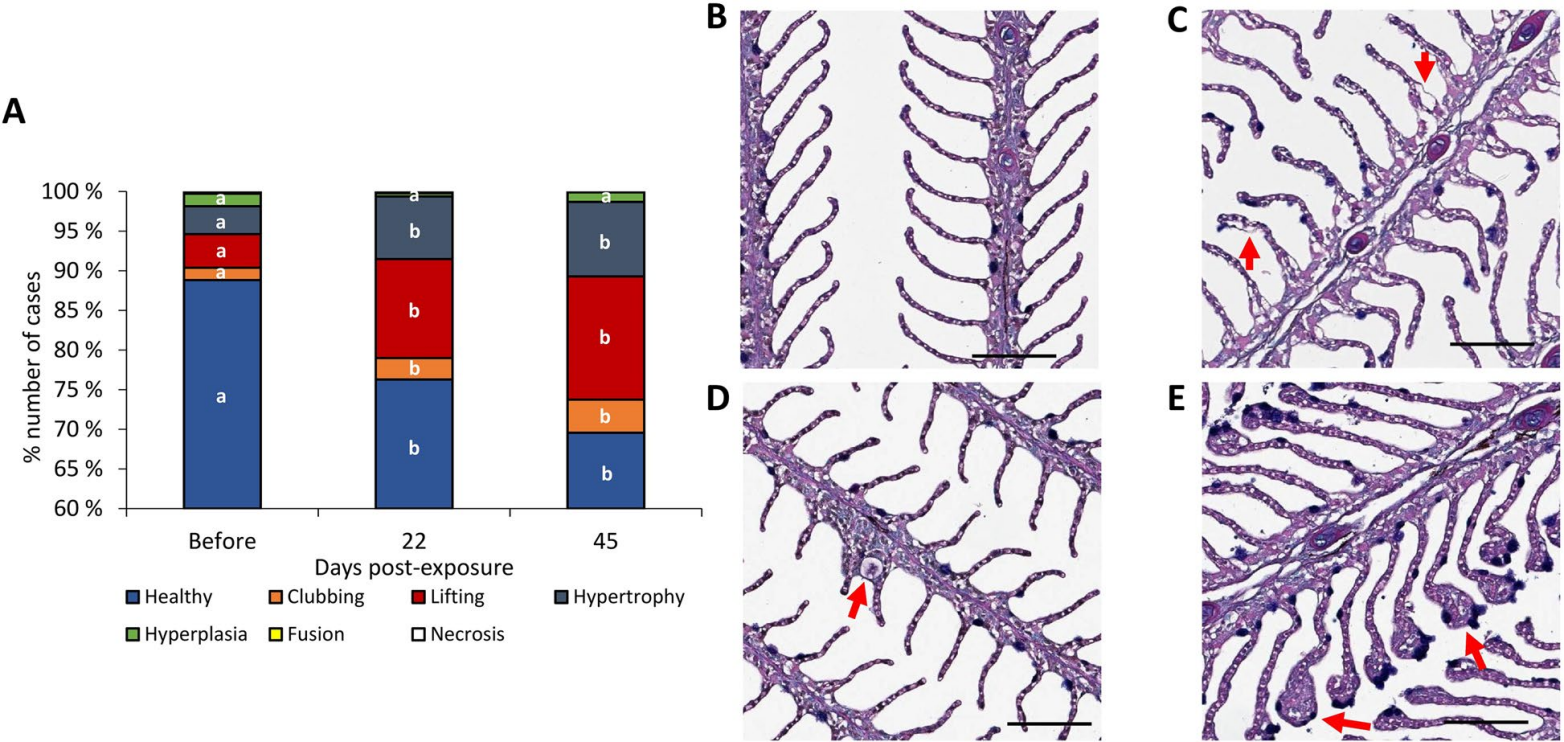
Quantification of histopathological cases in the gills of Atlantic salmon intermittently exposed to PAA. **A** Distribution of cases relative to the total number of lamellae evaluated. Seven key indicators were evaluated – unaffected, lamellar clubbing, epithelial lifting, hypertrophy, hyperplasia, lamellar fusion and necrosis. Values presented are mean ± SE of 8 individual fish per sampling point. Different letters denote significant difference at *P* < 0.05. Samples were collected before and 22 and 45 days after exposure. Representative photomicrographs showing **B** area of normal gills where no changes were observed, common in the pre-exposed fish, and 3 of the most common histopathological reversible changes quantified including **C** lifting, **D** hypertrophy, **E** clubbing as shown by arrowheads. Sections were stained with AB/PAS. Scare bar = 100 μm

Gill interlamellar space significantly decreased at day 45 of intermittent exposure compared with pre-exposure and at day 22 (Table 1). In addition, a progressive increase was observed throughout the trial in gill lamellar length. The total number of mucous cells at day 45 post-exposure was significantly higher compared with pre-exposure and at day 22 post-exposure. The same pattern was observed for the number of acidic mucous cells, but not for the number of neutral mucous cells. Quantitative histopathology of the gills revealed that almost 90% of the evaluated lamella were normal at the beginning of the trial (Fig. 6A) but decreased significantly to 70-75% following intermittent oxidant application. From the 6 pathological cases evaluated in the gills, a significant increase was observed for the number of epithelial lifting (Fig. 6A,C), hypertrophy (Fig. 6A, D), lamellar clubbing (Fig. 6A, E) following intermittent exposure of PAA, where the highest number of cases was recorded at day 45.

**Fig. 7:**
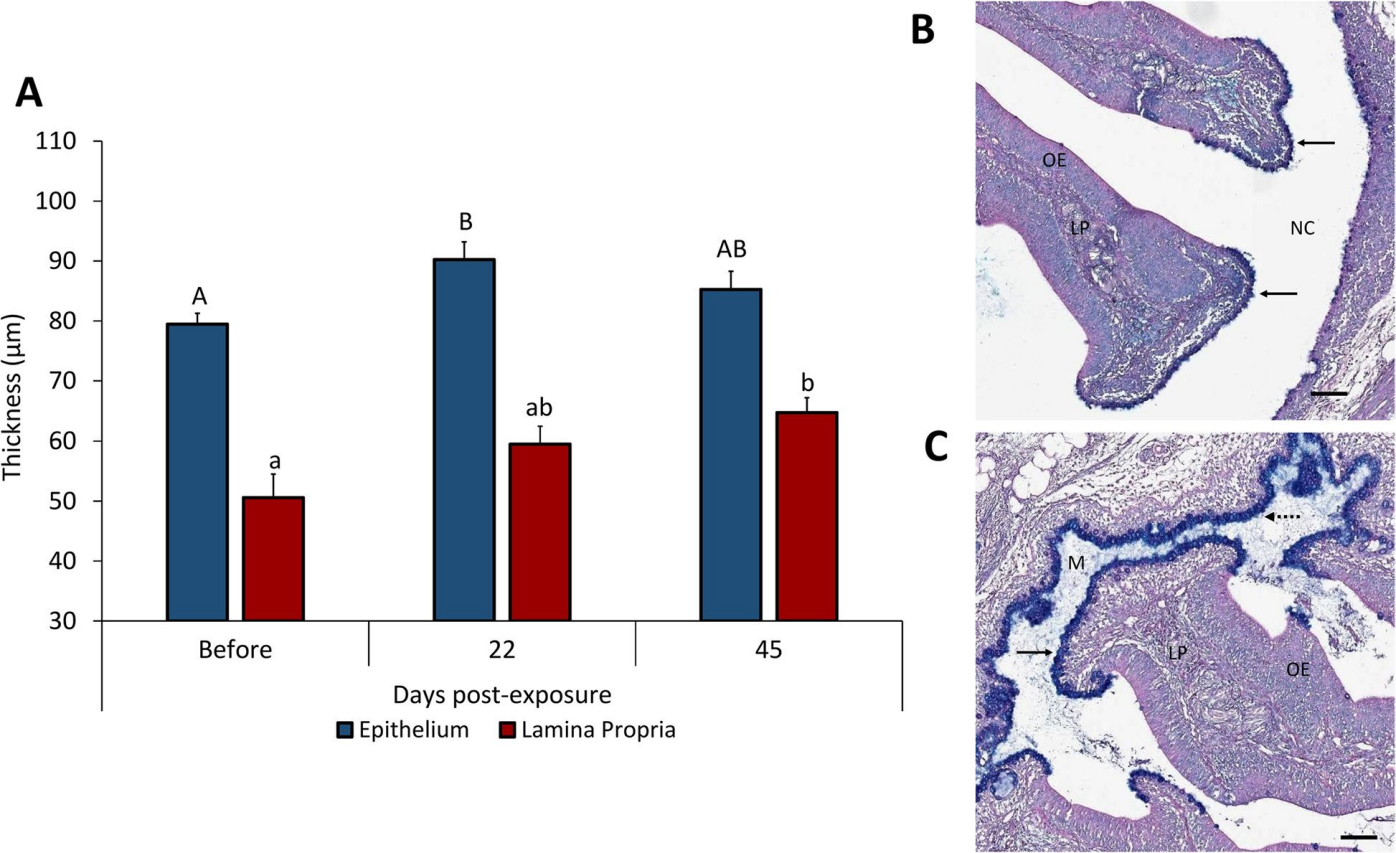
Histomorphological features of the olfactory rosette of Atlantic salmon intermittently exposed to PAA. **A** The thickness of the olfactory epithelium and *lamina propia*. Values presented are mean ± SE of 8 individual fish per sampling point. Different letters denote significant difference at *P* < 0.05. Samples were collected before and 22 and 45 days after intermittent oxidant exposure. **B** Representative pictographs of the olfactory rosette stained with AP/PAS showing a considerable increase in the mucous cells (arrow; stained dark blue) at the tip of the olfactory lamella at day 0 (**B**) compared with at day 45 (**C**). NC = nasal cavity; LP = lamina propia; OE = olfactory epithelium; M = mucus. Scale bar = 100 μm

The thickness of the olfactory epithelium significantly increased from pre-exposure to day 22 post-exposure (Fig. 7A). Nonetheless, the measurement at day 45 did not significantly vary between the two earlier time points. The *lamina propria* thickened through time where it was substantially thicker at day 45 compared with pre-exposure. Though no measurements were performed because of the difficulty and impractically to differentiate individual mucous cells, two impartial evaluators inferred that there was a clear tendency that mucous cells on the tip of the olfactory lamellae (Fig. 7B) became denser following intermittent application of PAA (Fig. 7C). In addition, mucous cells were predominantly concentrated on the tip of the olfactory lamella and the walls of the nasal epithelium.

### Physiological responses to a secondary stressor

**Fig. 8:**
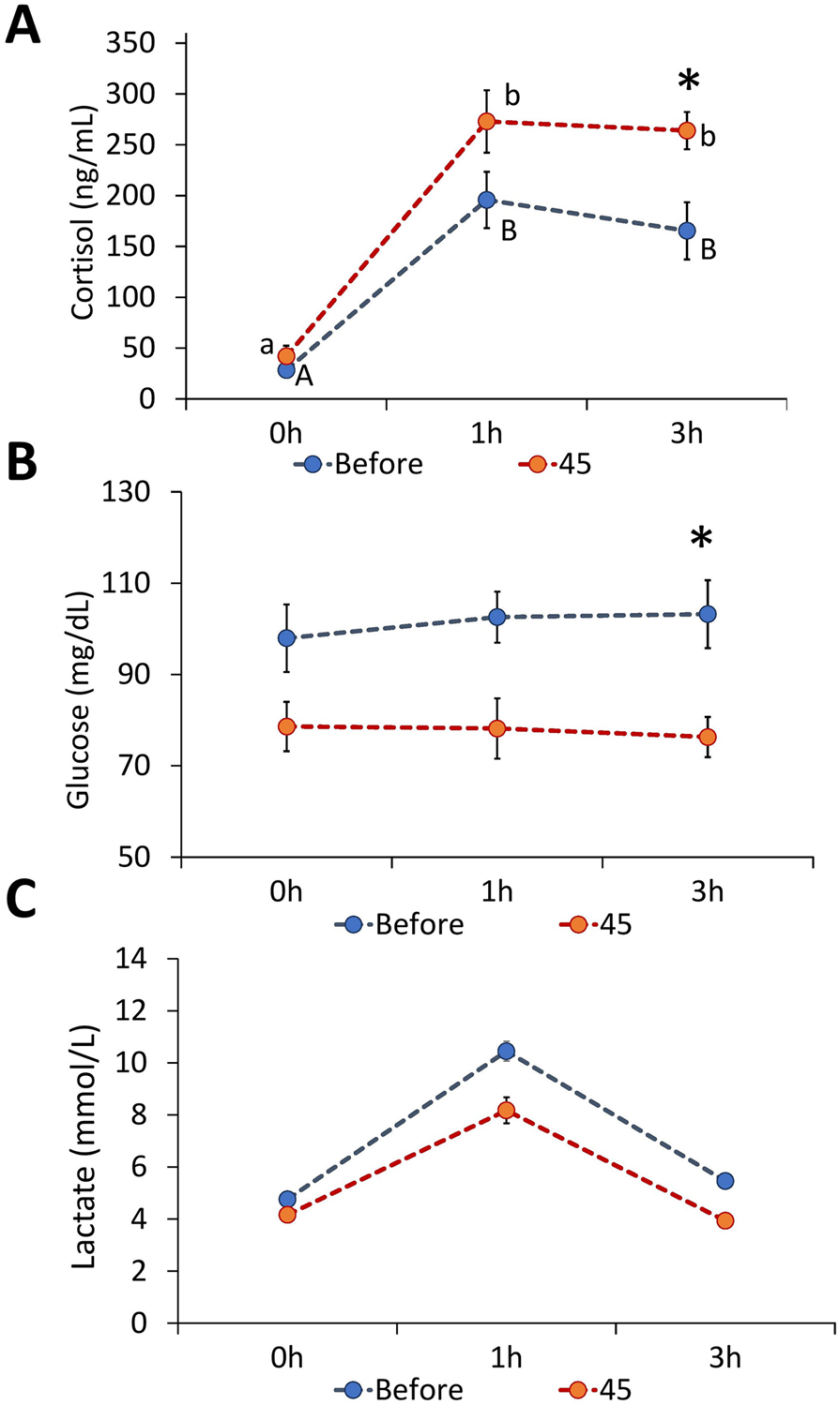
Stress parameters (cortisol, glucose and lactate) in the plasma of Atlantic salmon subjected to handling-confinement stress. The stress test was performed before the start of PAA application and at 45 days thereafter. Values presented are mean ± SE of 8 individual fish per sampling point. Different letters denote a significant difference. Asterisk (*) indicates a significant difference between two groups at a particular timepoint. Note that in cortisol, uppercase was for the group before exposure while lowercase was for the group at day 45 of intermittent exposure

After the handling-confinement stress test, the plasma cortisol level increased in the pre-exposed group as well as in the fish group intermittently exposed to PAA for 45 days (Fig. 8A). Plasma cortisol exhibited a 5.9-fold increase in pre-exposed fish, while an increment of around 5.5-fold was observed in the PAA-exposed group 1 h after the stress was induced. After 3 h, the cortisol level in both groups remained elevated compared with time 0. Comparing the two groups timepoint-wise, the elevated cortisol level in both groups at 1 h after stress was not significantly different. However, at 3 h after stress, PAA-exposed fish exhibited a significantly higher cortisol level than the non-PAA exposed fish.

Plasma glucose levels were unchanged following exposure to handling-confinement stress in both groups (Fig. 8B). It was apparent that the glucose level in non-PAA exposed fish was relatively higher than the group exposed intermittently to PAA, especially at 3 h post-stress, where a significant difference was identified.

Handling-confinement stress did not alter the plasma lactate level in the pre-exposed or intermittently-exposed salmon (Fig. 8C).

## Discussion

The application of PAA as a routine disinfectant in aquaculture production should be supported by a holistic understanding of how it influences the health and welfare of fish. To the best of our knowledge. This is the first report documenting the morphological and physiological impacts of intermittent PAA exposure in Atlantic salmon post-smolts reared in brackish water RAS. The systemic and mucosal changes indicate that the fish mobilised a network of physiological defences to counteract the risks from an oxidant-rich environment. Production performance was not affected by the treatment since no mortality, and altered feeding patterns were documented.

### Atlantic salmon mounts physiological stress responses to intermittent oxidant exposure

Increased plasmatic cortisol is a classic response to a stressful event in fish [30, 31]. Elevated cortisol levels were reported in rainbow trout and carp (*Cyprinus carpio*) after exposure to PAA, followed by negligible change after repeated pulse exposures, suggesting a form of habituation to the oxidant [24, 32]. In another study [10], carp intermittently exposed to PAA at 1 ppm showed a significant and persistent reduction of cortisol levels compared to the unexposed fish, where the authors implied a stress-protection role of PAA. However, this reduction of plasmatic cortisol did not reach baseline cortisol levels, suggesting chronic mild stress resulting from long term exposure to PAA in carp. In a recent study in salmon, a 30-min single exposure and re-exposure to PAA (i.e. 0.6 – 4.8 ppm) increased plasma cortisol levels [27]. However, no significant changes in plasma cortisol levels were observed in the present study following intermittent low-dose PAA exposure. Moreover, observed mean cortisol levels in the present study agreed with reported baseline levels for salmon [33]. The data, particularly 24 h after each treatment, indicate that intermittent low-dose application of PAA was not strong enough to trigger cortisol response, or salmon may have habituated to the treatment.

Plasma glucose levels increase to meet increased energy demands during stress [34]. It has been reported that increased cortisol levels modulate glucose production by enhanced glycogenolysis and gluconeogenesis during stress [34, 35]. The plasma glucose level remained unchanged during the first 2 weeks of exposure, implying that no significant stress-related energy mobilisation. This observation corroborates a previous study [27], where exposure to PAA at 0.6 and 2.4 ppm did not alter the glucose levels at 2 h, 48 h and 2 weeks post-exposure compared to pre-exposure levels. Interestingly, a significant decrease in glucose levels was observed in our study at days 22 and 45 post-exposure relative to pre-exposure level. This decrease may suggest that PAA induced hypoglycemia, however, it must be noted that the T0 level was at the upper threshold of the normal level, which skewed the temporal profile of plasma glucose. We could not fully establish the cause of a quite high glucose level at T0, but the measured glucose levels were within the normal range for salmon [34]. The temporal profile of glucose demonstrated an opposite trend compared with the temporal profile of TAC (Figs. 1C and 2A). In the mammalian model, it was shown that hypoglycemia induced changes in the oxidative stress markers [36], indicating a regulatory connection between these two physiological systems. It would be interesting to explore in the future whether such interplay is also present in salmon, nonetheless, the trend in the present study indicates that it may have a role in the adaptive responses to PAA.

Lactate increases due to anaerobic metabolism during a stressful episode [34, 37]. In a previous study, exposure and re-exposure to PAA did not significantly affect the plasma lactate levels in salmon [27]. In the current study, the day 22 level was significantly higher than the day 45 level, but both time points were not significantly different from the pre-exposure level. The first half of PAA administration could have triggered a strong metabolic pressure which was eventually accommodated by salmon as it adapted to the environment occasionally spiked with an oxidant. Though we cannot directly relate these levels with the documented levels during the first 2 weeks of administration (i.e. use of starved and not starved fish), the increasing tendency during these 2 weeks somehow provided an insight that lactate may have provided energy fuels to the stress adaptations in the early days of PAA administration.

Starvation or fasting evacuates the gut and reduces metabolism, oxygen demand and waste production, and in turn [38], reduces physiological stress during husbandry manipulation [39]. The reduction in metabolism is a protective mechanism to cope with fasting [40]. In this study, the samples collected from the 3 main sampling points had not been starved, while samples collected from the first 2 weeks were subjected to fasting. One reason why fasting was not performed during the routine samplings in the first 2 weeks was to avoid the confounding factor from periodic fasting. We expect metabolic differences between the two groups, given the influence of post-prandial processes on these measured variables. Therefore, no direct comparisons were made, and both sampling groups were treated as two independent sample groups.

### Atlantic salmon mobilise systemic antioxidant defences

PAA is a known source of exogenous reactive oxygen species (ROS), which naturally degrade but can be harmful to lipids, proteins and DNA molecules [37]. Thus, PAA-based disinfectants can prompt a transitory state of oxidative stress in exposed fish before full decay is achieved [26]. Antioxidants could scavenge ROS and prevent cellular oxidative stress. Elevated plasma total antioxidant capacity (TAC) indicates the mobilisation of antioxidants to counteract redox imbalance following oxidative stress [27]. Elevations in serum TAC levels by 8-fold and 5-fold were reported in rainbow trout following intermittent and continuous PAA exposure compared with unexposed trout [26]. Furthermore, increased TAC levels were documented in salmon after a re-exposure to PAA [27]. TAC levels showed a significant elevation at days 22 and 45 post-exposure in the present study compared with pre-exposure levels. This increase suggests that exposure to PAA led to an internal redox imbalance, likely triggering the activation of systemic antioxidants to scavenge excess ROS and maintain redox homeostasis. The elevated level is perhaps a protective mechanism against PAA-induced oxidative stress.

### Intermittent oxidant exposure differentially modulates the expression of immune and stress-related genes in mucosal organs but not in the liver

Mucosal surfaces in fish are a crucial first line of defence against the constant changes in the aquatic environment [41, 42]. Besides acting as a physical barrier, mucosal tissues play a vital role in teleost immunity [41–43]. Due to its permanent and intimate contact with the external environment, mucosal surfaces are highly responsive to environmental changes, characterised by transcriptional and proteomic changes and phenotypic alterations [42]. In the present study, significant modulation of gene expression in the mucosal organs was predominantly marked by a decrease in transcript levels, indicating that periodic oxidant exposure inhibited the defence mechanism at the mucosa or could also be related to potential habituation to intermittent exposure.

PAA exposure significantly downregulated the expression of *cu/znsod* and *gr* in the gills, a tendency previously reported [27, 44]. *Cu/znsod* catalyses the dismutation of superoxide radicals to H_2_O_2_ and O_2_ to neutralise oxygen radical-mediated toxicity [45]. The presence of H_2_O_2_ in PAA-based products could partly explain the decreased expression of *cu/znsod* in the gills since high levels of H_2_O_2_ can inhibit SOD activity [44, 46]. *Gr* is responsible for the regeneration of reduced glutathione, a crucial step in cellular antioxidant protection [47]. In the skin, upregulation of *gr* expression was observed, an opposite profile compared with the gills. These contradicting patterns of *gr* expression indicate that glutathione-mediated response was likely distinct between the gills and skin. As for the olfactory rosette, decreasing expression of *cat* was observed. *Cat* catalyses the transformation of H_2_O_2_ into O_2_ and water when present at high concentrations [48], and intermittent exposure likely interfered with this process. The responsiveness of antioxidant genes *gr*, *cu/znsod* and *cat* corroborates their known role in oxidative stress response [49, 50]. The observed changes suggest their crucial function in protecting the mucosa against PAA-induced oxidative damage. Nonetheless, no drastic and substantial changes occurred in the overall expression profiles of the antioxidant genes in the three mucosal tissues, implying that intermittent PAA exposure triggered minimal oxidative stress at the mucosal level.

Interleukins are a subset of cytokine molecules involved in the intercellular regulation of the immune system [51]. *IL-1β* is a pro-inflammatory cytokine, responsible for the mediation of the inflammatory response and cell proliferation [45]. Conversely, *il10* is an anti-inflammatory cytokine that inhibits macrophage activation, T-cell proliferation, and the production of pro-inflammatory cytokines [52, 53]. The expression of these interleukins can be induced by multiple stressors [51, 53]. The decrease in the expression of *il-1β* and *il10* observed in the gills indicates that PAA may interfere with the inflammatory process in response to an oxidant by inhibiting two crucial molecular regulators. These cytokines likely played a role in the progression of lesions in the gills as documented histologically.

*Hsp70* acts as a molecular chaperone and facilitates the repair and elimination of altered or denatured proteins, whereas *hsp90* has an essential role in supporting various components of the cytoskeleton, enzymes and steroid hormone receptors [54, 55]. In the present study, a transient decrease of *hsp90* expression was observed in the gills, while in the olfactory rosette, the expression of *hsp70* and *hsp90* decreased after intermittent exposure to PAA. There are two potential explanations as to why the decrease in expression. First, even though PAA triggered oxidative stress, it was minimal to initiate countermeasures from *hsp*s. On the other hand, the decrease in expression may be associated with the diminishing response to repeated encounters with an oxidant, which further suggests a form of desensitisation or habituation.

Mucins are the main components of the mucus and are high molecular weight, filamentous and highly glycosylated glycoproteins, playing a crucial for mucosal defence [56, 57]. In humans, oxidative stress is proposed to upregulate the production and secretion of mucin glycoproteins in airway mucus, namely MUC5AB and MUC5B, which are linked with hyperplasic and hypertrophic mucous cells in the airway epithelium [58]. Intermittent exposure to PAA only affected the transcription of gel-forming mucins (i.e., *muc5ac*, *muc5b* and *muc2*) in the gills and skin, where a general profile displayed transient downregulation mid-way and then returned to the pre-exposure level at termination. The oxidant had no direct impact on the phenotypic properties of the mucous cells; however, the effects were more striking on the biochemical property, such as in mucin expression. In addition, the overall profile suggests a potential recovery following a transcriptional dampening midway through the exposure. Despite a noticeable change in the mucous cell population of the nasal olfactory mucosa, none of the mucin genes in the study were markedly affected. This indicates that PAA effects in the nasal mucosa were mainly at the phenotypic (i.e. more mucous cells to protect the mucosa from an irritating oxidant) and not considerably at the biochemical level, quite distinct compared with the gill and skin profiles. Since mucin is a large class of glycopolymeric proteins, there are perhaps other mucins that were affected but not covered in the present study.

The liver is an important organ in xenobiotic metabolism, and as such, may play a role in the organisms’ responses to oxidants in the environment. However, in the present study, no remarkable changes were observed in the expression of selected genes in the liver, indicating that the impacts of intermittent PAA exposure on the stress and immune-related genes were mainly at the mucosa.

### Key structural features of the mucosal organs are altered by intermittent oxidant exposure

No significant pathological alterations were identified in the skin in any of the sampling points. However, the observed higher scores for epithelial surface quality following intermittent oxidant exposure suggest that exposure might have somewhat compromised the epithelial surface of the skin. The observed increase in dermal thickness at 45 days of exposure could act as a compensatory mechanism of the skin, providing additional protection from diffusion/uptake of PAA and H_2_O_2_ despite the slightly compromised epithelial surface. Another possibility is that the observed increase in dermal thickness is a consequence of salmon development [59]. A correlation was found between dermal thickness and weight (*P* < 0.001, *r* = 0.71) and length (*P* < 0.001, *r* = 0.71). Overall, these results are supported by previous studies where no significant histostructural changes in the skin were observed after different regimens of PAA exposure [26, 29].

The gills are a multi-purpose organ responsible for respiration, maintaining optimal osmotic pressure and acid-base balance of body fluids. Because gills are in direct contact with the water, they are particularly vulnerable to various injuries [60, 61]. The decreased interlamellar space at day 45 was most likely the result of hyperplasia, hypertrophy and oedema cases in the base of the lamellae. Although these alterations may act as a protective and adaptive mechanism by augmenting the oxidant diffusion distance, the respiratory surface becomes reduced; as a result, impairing gill function to some extent [62]. Increased mucous cell numbers have been documented in response to persistent gill irritation [60, 61]. The concurrent increase in the number of acidic mucous cells may reflect a defence mechanism since higher proportions of acidic mucous cells are linked with an increase in the viscosity of mucus that helps to prevent chemical damage to the epithelium [63]. Currently, conflicting data on gill histopathological alterations due to PAA exposure exists [10, 26, 44], likely resulting from different parameters between the conducted studies and the composition of PAA trade products [29]. In our study, even though the highest number of lesions was observed at the end of the exposure, only epithelial lifting, hypertrophy and lamellar clubbing, all reversible lesions [60], showed a significant increase following PAA exposure. Therefore, it seems that intermittent oxidant exposure led to some reversible pathological changes in the gills. The overall gill health status was not severely compromised. If given enough time, the fish would likely show a recovery from the reported lesions.

In salmonids, the olfactory epithelium lines a multi-lamellar olfactory rosette which is covered by sensory and non-sensory epithelium [64]. The sensory epithelium is susceptible to water contaminants [65]. The olfactory rosette of rainbow trout contains abundant myeloid and lymphoid cells, which has a strong capacity to mount innate and adaptive immune responses [43]. In the present study, the documented enlargement of the epithelium and *lamina propria* could act as an improved barrier protection against oxidant uptake to safeguard the olfactory system’s integrity and function. Moreover, because of more external positioning, the epithelium presents an earlier point of contact than the *lamina propria* to the oxidant. A significant increase in the secretion of olfactory mucus is described in response to the presence of various chemical compounds. Thus, the perceived increase in the density of mucous cells at the tip of olfactory lamellae likely promotes improved protection of the olfactory sensory neurons and regulation in detecting external cues and chemosensory responses [66].

### Intermittent oxidant exposure elicits a minimal interference on the physiological responses to handling-confinement stress

Exposure to toxicants or chemical pollutants is known to potentially cause exhaustion of the pituitary-interrenal axis and consequently impair the ability of the fish to increase cortisol in response to an acute stressor [30]. On the other hand, chronic exposure to mild stressors may also desensitise fish and mitigate the neuroendocrine and metabolic responses to acute stressors [31]. Gesto et al. [25] reported that both unexposed and PAA-exposed (intermittently or continuously) rainbow trout exhibited increased plasma cortisol levels after chasing stress, revealing that PAA exposure did not change the typical cortisol response when prompted with a secondary stressor. In the present study, the cortisol response following handling and confinement demonstrated a similarly elevated level in plasma before and after intermittent oxidant exposure. Interestingly, the cortisol level at 3 h was significantly higher in the group intermittently exposed to PAA than the unexposed group. These observations suggest that previous intermittent exposure to PAA did not dramatically alter the ability of fish to mount a classical cortisol response to an acute secondary stressor. However, it may slightly influence the kinetics of cortisol recovery, which is interesting to explore in future studies. Glucose levels following exposure to handling-confinement stressors did not significantly change before and after PAA exposure. It is important to note, however, that the post-stress glucose level of PAA-exposed fish was relatively lower than the non-PAA-exposed fish. This indicates that PAA administration minimally interfered with glucogenesis which may eventually influence the mobilisation of energy supply following stress. This partly provided insight into the lower glucose level observed in T22 and T45 of the PAA administration. Overall, the influence of PAA application to post-stress responses was minimal, however, the slight interference in the kinetics and magnitude of responses should be explored further in the future, especially by extending the duration of post-stress analysis to understand the recovery process.

## Conclusions

The study revealed that intermittent exposure of salmon to PAA, a strong oxidant, initiated physiological and histostructural changes, underscoring both mucosal and systemic responses. No drastic changes were observed in the plasmatic levels of the classical stress indicators, implying that PAA provoked low levels of systemic stress. PAA seemed to cause an internal redox imbalance leading to systemic oxidative stress, which was compensated with increased production of systemic antioxidants. Intermittent oxidant exposure differentially affected several genes encoding for antioxidants, cytokines, heat shock proteins and mucins - where downregulation was the prominent profile in the gills and olfactory rosette, whereas upregulation was apparent in the skin. Such a distinct profile indicates that mucosal organs responded differently to PAA, which may be an adaptive mechanism for coordinating a robust mucosal response to the oxidant. While PAA led to varying levels of histostructural alterations in the three mucosal organs, the gills were considerably affected, with reversible pathological lesions increased following intermittent oxidant exposure. PAA did not dramatically alter the ability of salmon to mount a physiological stress response in the presence of a secondary stressor though some subtle interference was documented, indicating that though PAA is generally a welfare-friendly antimicrobial oxidant, attention must be given to the influence of application frequency. The data presented here underline the biological consequences of PAA in salmon, where the overall profile demonstrates that while it presents physiological pressures as a potential mild stressor, the fish were able to coordinate an interconnected response likely in the form of adaptation and habituation to its presence in the rearing environment. These results lend support to the potential application of PAA as a routine disinfectant in salmon RAS production.

## Methods

### Intermittent exposure to a peracetic acid-based oxidant

The study presented herein was conducted in a semi-commercial scale RAS at Nofima Centre for Recirculation in Aquaculture (NCRA) in Sunndalsøra, Norway, simulating the use of PAA in a typical production scenario. The data were discussed within a pre-exposure/post-exposure context. Seven hundred and thirty-five fish (735, starting weight *ca* 90 g, Bolaks strain) were randomly transferred to each of the 4 × 3.2m^3^ octagonal tanks connected to a recirculating system composed of a microscreen belt filter, a moving bed bioreactor, a degasser column, and two holding sump units. The system was running under the following operational parameters: RAS water volume = 41 m^3^, average total flow = 534 L min^− 1^, tank water volume = 3.2 m^3^, tank water flow rate 100 L min^− 1^, retention time = 32 min, daily water exchange = 20%. The initial density in the tank was *ca* 20 kg/m^3^. Additional technical specifications of the system are described in an earlier publication [67]. Fish were allowed to acclimatise for 3 weeks under the following conditions: salinity at 11.6 ± 0.5 ‰, the temperature at 12.8 ± 0.6 °C, pH at 7.5, dissolved oxygen > 90% saturation and photoperiod set at 24 h light. Similar conditions were followed throughout the exposure trial. In addition, the levels of ammonia (NH_4_–H: 0.093 ± 0.1 mg/L), nitrate (NO_3_–N: 9.54 mg/L) and nitrite (NO_2_–N: 0.024 ± 0.02 mg/L) were analysed 3 times a week and maintained at safe thresholds. During the trial, fish were fed daily over 24 h with a commercial diet (Nutra Olympic 3 mm, Skretting, Averøy, Norway: Proximate composition: Moisture 8%, Crude fat 23%, Crude protein 49%, Ash 10%) administered through a belt feeder.

A peracetic acid-based disinfectant (Perfectoxid, PAA) was supplied by Novadan ApS (Kolding, Denmark). After the acclimation period and the pre-exposure samples were taken, PAA was directly applied to each tank at a final concentration of 1 mg L^− 1^ every 3 days for 6 weeks, making 15 applications in total. This concentration and mode of delivery were patterned on a previous PAA experiment conducted in trout, a closely related species of salmon [26]. The product was administered between 0900 and 1000 AM to avoid temporal effects of PAA [68] and at four different locations in the tank to ensure proper distribution. The predicted exponential decay of PAA in brackish water is around 0.020-0.030 h^− 1^ [21]. The behaviour of the fish was monitored daily by visual inspection.

### Stress test

A stress test composed of handling and confinement was performed 4 days before the first PAA application (pre-exposure response) and at day 45 of the intermittent PAA exposure (post-exposure response). Fish were starved for 24 h before the test. Before the stress test was performed, ten fish were netted out from each tank, humanely euthanised with an overdose of Tricaine methanesulfonate (MS-222), and blood was collected from the caudal artery by a heparinised vacutainer (BD Vacutainer™, USA). This group of fish served as the pre-stress fish or T0. The handling-confinement stress protocol was as follows: 20 fish per tank (*N* = 80, in total) were randomly dip-netted, exposed to air for 15 s, confined in a bucket for 5 min to achieve a density of *ca* 230 kg m^3^ and after that transferred to a recovery tank with aeration (DO > 90% saturation). Each experimental tank had its corresponding recovery tank. Post-stress blood collection was performed at 1 (T1) and 3 (T3) hours after the stress test with a similar collection protocol described for T0. Ten (10) fish were taken from each recovery tank at each post-stress sampling. Plasma was separated from the blood by centrifugation for 10 min at 5200 rpm and thereafter stored at − 80 °C until analysis.

### Sample collection

Three comprehensive tissue samplings were performed: before exposure, 22 days (3 days after the 7th addition) and 45 days (3 days after the 15th addition) after intermittent PAA exposure. Feeding was restricted 24 h before sample collection. Ten fish were randomly taken from each tank and humanely euthanised with an overdose of MS-222. Following length and weight measurements, the external welfare scoring was executed as previously described [69]. To ensure objectivity, only one person performed blind scoring of all individuals throughout the trial. Plasma was collected following the protocol described in Mucosal and hepatic expression of selected immune and stress-related genes section and stored at − 80 °C until analysis. A section of dorsal skin (just below the dorsal fin), the second gill arch, the olfactory rosette and liver were dissected and divided into two portions. A fraction of the tissues were suspended in RNAlater™ (Ambion, USA), kept at room temperature overnight to allow penetration and then stored at − 80 °C until RNA extraction. The remaining dorsal skin, olfactory rosette and second gill arch were stored in 10% neutral buffered formalin (BiopSafe®, Denmark).

To follow the systemic responses of salmon during the early phase of oxidant administration, plasma was collected from 5 fish per tank 24-h after each PAA application within the first 2 weeks in a similar manner as described above, though they were not starved prior to sampling. We acknowledge the post-prandial influence on these measured variables, therefore comparisons were restricted within these 2 weeks. All samples were collected in the same period during each occasion (0900-1000 AM) to avoid the influence of circadian rhythm in these parameters. Samples were stored at − 80 °C until analysis.

### Plasma stress indicators

Three commercially available colourimetric assay kits were employed to determine the levels of the key plasma stress indicators. Plasma cortisol was quantified using a solid-phase Enzyme-linked Immunosorbent Assay (ELISA) Kit (Demeditec Diagnostics GmbH, Kiel, Germany) following the manufacturer’s instructions. Plasma glucose was determined using a Colorimetric Detection kit (Arbor Assays, Michigan, USA). Plasma lactate was analysed using a Lactate Assay Kit in Pentra C400 (HORIBA ABX, Montpellier, France). Total antioxidant capacity (TAC) was determined using the Total Antioxidant Capacity Assay Kit (Sigma-Aldrich, USA) as previously verified in salmon [27]. All samples were analysed in duplicates.

### Histological processing and assessments

The gills, skin and olfactory rosette tissue samples kept in formalin were embedded in paraffin following a 10-h long processing programme of 70, 90, 90, 96% and 3 × 100% ethanol, 3x xylene and 2x paraffin (Leica TP1020, Germany). Paraffin-embedded tissue samples were cut into 5 μm section using a rotatory microtome (Leica RM2165, Germany), placed onto microscope slides, heat-fixed at 60 °C overnight, dehydrated, and stained with Periodic Acid Schiff-Alcian Blue (AB/PAS) in an automated stainer (ST5010, Germany) and photographed using a digital slide scanner (Aperio CS2, USA).

Histological evaluation of the gills was performed at 8 randomly selected locations of the whole gill arch. Each field contained 40 lamellae, accounting for a total of 320 lamellae investigated per fish. Mucous cells were quantified at the filament and the lamellae and differentiated as either acidic (bright blue) or neutral (magenta) mucous cells. Lamellar length (measured from base to the tip) and interlamellar space (measured from the base of one lamella to another) were measured. Moreover, quantitative histopathology was performed following a previously published method [28, 70]. Six key branchial histopathological changes were identified, including lamellar clubbing, epithelial lifting, hyperplasia, hypertrophy, lamellar fusion and necrosis. A lamella that did not show any sign of damage or lesion was defined as “healthy”. Moreover, descriptive histopathology was performed by an impartial evaluator to assess the overall quality of the gill tissue.

For the skin, measurements were carried out in 3 randomly selected regions of *ca* 500 μm in the distance per area. In each region, epidermal mucous cells were counted and defined as either acidic or neutral mucous cells. Epidermal and dermal thickness was also measured in 5 different locations of the selected region. The microscopic general appearance of the epidermis and the quality of the epithelial surface were characterised using the semi-quantitative 3-point scale skin health scoring system [28, 29].

For the olfactory rosette, measurements were taken from 3 randomly selected olfactory lamellae in each fish. The thickness of the olfactory epithelium and *lamina propria* were systematically measured in 5 distinct locations in the mid-region of the olfactory lamellae to ensure uniformity. Because of the high density per unit area (see Fig. 7), it was challenging to have an impartial and structured counting strategy in the number of mucous cells thus, we opted for descriptive evaluation from two evaluators.

### RNA isolation, cDNA synthesis and qPCR assay

Total RNA was isolated from the gills, skin, olfactory rosette and liver using Quick-RNA™ Microprep Kit (Zymo Research, USA). RNA concentration and quality were determined using a NanoDrop 8000 spectrophotometer (Thermo Scientific, USA). Complementary DNA (cDNA) was synthesised by reverse transcription using Taqman Reverse Transcription Kit (Applied Biosystems, USA) in a 20 μL reaction mixture containing 9.6 μL 500 ng template RNA, 2 μL 10X RT Buffer, 1.4 μL 25 mM MgCl_2_, 4 μL 10 mM dNTP mix, 1 μL RNase Inhibitor, 1 μL MultiScribe™ Reverse Transcriptase and 1 μL Random Hexamers. Thermocycling was performed using a Veriti™ 96-Well Thermal Cycler (Applied Biosystems, USA), and the parameters were as follows: 25 °C for 10 min, 37 °C for 30 min and 95 °C for 5 min.

The transcript levels of selected genes were quantified by real-time quantitative polymerase chain reaction (RT-qPCR) in QuantStudio™ 5 Real-Time PCR System (Applied Biosystems, USA). Each assay consisted of 5 μl of PowerUp™ SYBR™ Green Master Mix (Applied Biosystems, USA), 0.5 μl 10 μM of each forward/reverse primer (Invitrogen, USA) and 4 μl of 1:10 cDNA. The cycling parameters were as follows: pre-incubation at 95 °C for 20 s, amplification with 40 cycles at 95 °C for 1 s and 60 °C for 20 s, and a dissociation stage of 95 °C for 1 s, 60 °C for 20 s and 95 °C for 1 s. A five-step standard curve of 2-fold dilution series was prepared from pooled cDNA to determine the amplification efficiencies. Transcript levels were expressed as a relative expression after normalisation using the geometric mean of two reference genes (*Elongation factor alpha-1* and *β-actin*), as described previously [71]. These two genes were identified to be stably expressed in the samples after a preliminary trial that tested several housekeeping genes. The primers used in the study are provided in Table 2.

**Table 2:** Primers used in the present study

Gene name	Abbreviation	Sequences (5′ → 3′)	Reference
*Glutathione peroxidase*	*gpx*	F: GATTCGTTCCAAACTTCCTGCTA	[72]
		R: GCTCCCAGAACAGCCTGTTG	
*Glutathione reductase*	*gr*	F: CCAGTGATGGCTTTTTTGAACTT	[72]
		R: CCGGCCCCCACTATGAC	
*Glutathione S-transferase*	*gsta*	F: AGGGCACAAGTCTAAAGAAGTC	[68]
		R: GTCTCCGTGTTTGAAAGCAG	
*Manganese superoxide dismutase*	*mnsod*	F: GTTTCTCTCCAGCCTGCTCTAAG	[72]
		R: CCGCTCTCCTTGTCGAAGC	
*Copper/Zinc superoxide dismutase*	*cu/znsod*	F: CCACGTCCATGCCTTTGG	[72]
		R: TCAGCTGCTGCAGTCACGTT	
*Catalase*	*cat*	F: GGGCAACTGGGACCTTACTG	[73]
		R: GCATGGCGTCCCTGATAAA	
*Interleukin 1β*	*il1b*	F: AGGACAAGGACCTGCTCAACT	[53]
		R: CCGACTCCAACTCCAACACTA	
*Interleukin 10*	*il10*	F: GGGTGTCACGCTATGGACAG	[53]
		R: TGTTTCCGATGGAGTCGATG	
*Heat shock protein 70*	*hsp70*	F: CCCCTGTCCCTGGGTATTG	[72]
		R: CACCAGGCTGGTTGTCTGAGT	
*Heat shock protein 90*	*hsp90*	F: CCACCATGGGCTACATGATG	[74]
		R: CCTTCACCGCCTTGTCATTC	
*Mucin 5 ac-like*	*muc5ac*	F: GACCTGCTCTGTGGAAGGAG	[57]
		R: AGCACGGTGAATTCAGTTCC	
*Mucin 5b-like*	*muc5b*	F: ATTAAGAGCGATGTCTTCACAGC	[57]
		R: AAGCACATGAGTCTCTCACACAA	
*Mucin 2-like*	*muc2*	F: GAGTGGGCTCTCAGATCCAG	[57]
		R: GATGATGCGGACGGTAGTTT	
*Elongation factor alpha 1*	*ef1a*	F: GAATCGGCTATGCCTGGTGAC	[75]
		R: GGATGATGACCTGAGCGGTG	
*Β-actin*	*actb*	F: CCAAAGCCAACAGGGAGAA	[76]
		R: AGGGACAACACTGCCTGGAT	

### Data handling and treatment

A Shapiro-Wilk test was used to evaluate the normal distribution and an F-test to check for the equal variance of the data from plasma stress indicators, total antioxidant capacity, gene expression analysis and histological assessment. A one-way ANOVA was used to test for differences between exposure periods, followed by Tukey’s multiple comparison test when significant differences were observed. The Holm-Sidak test was used to identify pairwise differences. For the data of epidermal general appearance and surface quality, a Fisher’s Exact Test was performed. Statistical tests were executed using R studio (version 1.2.5019). The level of significance was set at *P* < 0.05, except when the Holm-Sidak test was performed, for which the significance level was set at *p* < 0.025. Values are expressed as mean ± SE.

## Supplementary Information

**Additional file 1.** Summary of welfare scores.

**Additional file 2.** The ARRIVE Essential 10: Compliance Questionnaire.

## Data Availability

The datasets used and/or analysed during the current study are available from the corresponding author on reasonable request.

## Abbreviations

PAA: Peracetic acid
H_2_O_2_: Hydrogen peroxide
RAS: Recirculating aquaculture system
TAC: Total antioxidant capacity
